# Prevalence and aetiology of coccidiosis in broiler chickens in Bejaia province, Algeria

**DOI:** 10.4102/ojvr.v85i1.1590

**Published:** 2018-09-18

**Authors:** Nedjima Debbou-Iouknane, Hama Benbarek, Abdelhanine Ayad

**Affiliations:** 1 Department of Environment Biological Sciences, University Abderrahmane Mira Bejaia, Algeria; 2 Department of Agricultural Sciences, University Mustapha Stambouli, Mascara, Algeria

## Abstract

The prevalence of coccidiosis was determined and *Eimeria* species were identified in farms at different locations in the Bejaia region, Algeria. The study was conducted from February to December 2016. Unvaccinated birds were selected randomly. Samples from litter and faeces were collected randomly (147 and 109, respectively). Necropsy and parasitological examinations were carried out using standard methods. Of the samples examined, 93 out of the 147 litter samples and 78 out of the 109 intestinal content samples were infected with *Eimeria* oocysts (63.26% and 71.55%, respectively). Mixed infections with *Eimeria* spp. were observed in some of the positive farms, with an overall prevalence of 54.28%. Five species of *Eimeria* (viz. *E. acervulina, E. tenella, E. maxima, E. brunetti* and *E. mitis*) were identified with different indices. *Eimeria acervulina* followed by *E. tenella* were the predominant species infecting chickens at the farms visited (32.05% and 26.92%, respectively). Statistically, the most prevalent *Eimeria* spp. was *E. Acervulina* (*p* < 0.05). This study demonstrated that coccidiosis is an omnipresent parasitic intestinal disease. It could strongly decrease production performance in broiler chickens.

## Introduction

In Algeria, the poultry sector represents a significant portion of the agricultural economy, with 9.84% of animal production (Rachid 2011). In addition, poultry breeding annually produces an average of 340 000 tons of white meat and over 4.8 billion eggs (Alloui & Bennoune 2013). According to the Provincial Direction of Agricultural Services, a large part of the poultry sector is concentrated in the northern provinces of the country, mainly in Bejaia. It is one of the best sources of high biological value animal protein. In the last years, the Algerian authority has adopted a policy to improve the livestock production sector through intensification of poultry production to satisfy consumer need and ensure food security.

Avian coccidiosis is defined as an enteric parasitic pathology caused by the protozoa *Eimeria* spp. It affects the epithelial cells of birds between the ages of 3 and 18 weeks (Nematollahi, Moghaddam & Farshbaf Pourabad 2009; Toulah 2007). Coccidiosis is one of the major causes of poor performance and productivity loss in poultry and other farm animals (Bachaya et al. 2012; Lin, Decuypere & Johan 2006; Mujahid, Akiba & Toyomizu 2007). This disease is endemic in most tropical and subtropical regions. It is favoured by ecological and management conditions (Obasi, lfut & Offong 2006).

Coccidiosis is recognised as the parasitic disease with the greatest economic impact on poultry industries worldwide (Allen & Fetterer 2002). It causes important production losses and there is a high cost treatment or prevention. (Shirley, Smith & Tomley 2005). According to Chapman (2009), coccidiosis may cost the US (United States) chicken industry about $127 million annually. Because of these huge economic losses, coccidiosis has become a major problem in poultry farms worldwide. It is pertinent to continually evaluate the prevalence and the management of this pathology. In Algeria, this disease is poorly documented (Triki-Yamani & Bachir Pacha 2010; Triki-Yamani et al. 2014). That is why it is difficult to assess its magnitude and impact on production costs. The objective of this investigation was to determine the prevalence of coccidiosis and to diagnose the *Eimeria* spp. on farms at different locations in Bejaia province, Algeria. The ultimate goal was to constitute a scientific basis for this disease in the country.

## Materials and methods

### Study area

**FIGURE 1:**
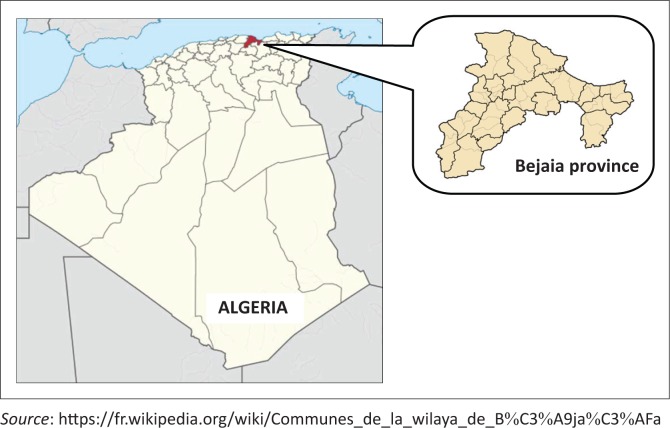
Map of the study area, Bejaia (Algeria).

The study was carried out in different localities of Bejaia province, Algeria (36°43’N, 5°04’W) (Figure 1). It has an area of 326 826 km^2^ with a poultry population of 3 291 050. The annual winter rainfall in the region averages 680 mm. The mean maximum summer temperature reaches 25.3 °C (August) and the mean minimum winter temperature falls to 13.4 °C (March). The monthly rainfall and mean temperature during the period of the present study are presented in Table 1.

**TABLE 1:** Annual rainfall and temperature data of the Bejaia area (Algeria) during 2016.

Month	Temperature (°C)			Rainfall (mm)
	Mean	Min	Max	
January	14.1	9.0	19.2	101
February	14.7	9.5	19.8	113
March	13.4	8.4	18.3	196
April	16.2	11.8	20.6	48
May	18.6	14.1	23.2	61
June	21.9	17.4	26.4	13
July	25.0	20.6	29.3	0
August	25.3	20.7	29.8	0
September	23.9	19.1	28.8	39
October	23.2	18.2	28.2	20
November	18.0	13.1	22.8	45
December	14.6	10.2	19.1	45

*Source*: https://www.infoclimat.fr/climatologie/annee/2016/bejaia/valeurs/60402.html

Min, minimum; Max, maximum; mm, millimetre.

### Animals and sampling

The study was conducted from February to December 2016. Thirty-five poultry farms were visited. Each farm has an average capacity of 2000 broiler chickens. The flocks were visited on the basis of abnormal mortality associated with lameness, diarrhoea, decreased feed intake and low body weight. Information regarding the age of birds, history of diarrhoea and other chicken characteristics was collected from farmers. The bird age selected was from 1 to 50 days old. Unvaccinated birds were selected randomly. Samples from litter and faeces were collected randomly (147 and 109, respectively). The faecal samples from the farms visited during the study period were collected from different locations in the pens to ensure repeatability. All samples were kept at +4 °C until analysis. Necropsy and parasitological examinations were carried out using standard methods at the Laboratory of Animal Biology, University of Bejaia.

### Sample examination

All the intestines and caeca were examined carefully for the presence of *Eimeria* oocysts and external lesions. The intestines were cut open using a scalpel blade (Lobago, Worku & Wossene 2005) and the gut contents were microscopically examined by flotation technique (Soulsby 1986). The positive samples were kept in a 2.5% aqueous solution of potassium dichromate (K_2_Cr_3_O_7_) for sporulation as described by Al-Quraishy, Abdel-Baki and Dkhil (2009). Counting of oocysts was done using the McMaster counting technique and expressed as per gram of faeces (OPG [oocysts per gram]) (Conway & McKenzie 2007; Haug et al. 2008). The lesion score was based on lesion severity from 0 to 4 on various intestinal parts (Johnson & Reid 1970).

### Identification of *Eimeria* species

The identification of *Eimeria* species in chickens was done on the basis of criteria such as size, shape, presence or absence of micropyle and its sporulation time (Carvalho et al. 2011; Long & Reid 1982; Soulsby 1982). The intestine localisation (McDougald 2003) and the gross appearance and characteristics of intestinal lesions (Johnson & Reid 1970) were also considered for this study.

### Histopathological examination

The tissue samples were collected in 10% buffer formalin (Sigma Aldrich, St. Louis, MO). The processing consisted of serial dehydration, clearing and impregnation with wax. Tissue sections 5 *µ*m thick were cut with a microtome and were fixed on slides. A staining procedure was carried out using haematoxylin and eosin (Sigma Aldrich, St. Louis, MO). The slides were examined with an optical microscope (Leica Microsystems GmbH, Wetzlar, Germany) (Luna 1968).

### Statistical analysis

The data were collected and analysed initially in Microsoft Office Excel 2007 to obtain the prevalence of coccidian oocysts. A statistical analysis was performed by using JMP^®^ Software, version 7.0 (SAS Institute Inc, 2007). The *t*-test was used to compare the prevalence of *Eimeria* spp. The coccidiosis prevalence was analysed using age (1–10 days, 11–20 days, 21–30 days, 31–40 days and 41–50 days) as a factor of variation. The statistical analysis was performed using analysis of variance (ANOVA). The values were statistically significant when *p* < 0.05.

## Results

Nineteen farms out of 35 visited were infected by *Eimeria* spp. In all, 93 out of 147 litter samples and 78 out of 109 intestinal content samples were infected with *Eimeria* oocysts (63.26% and 71.55%, respectively). Mixed infections with *Eimeria* spp. were observed in some of the positive farms, with an overall prevalence of 54.28%.

**FIGURE 2:**
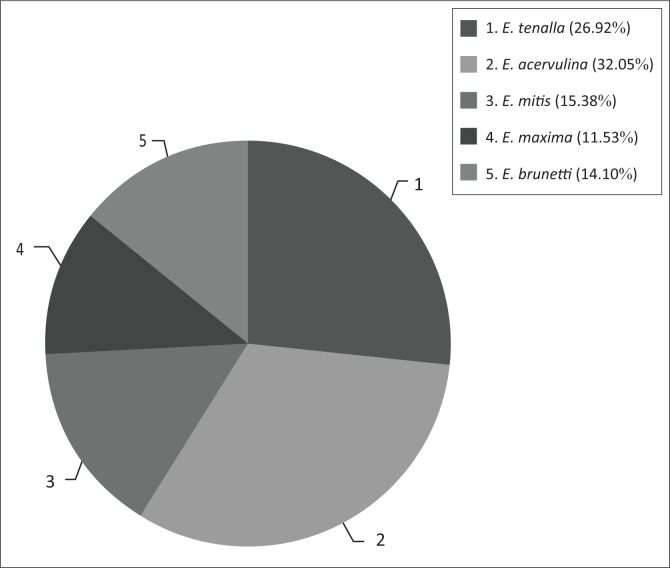
Prevalence of various *Eimeria* spp. at broiler chicken farms in the Bejaia area.

Species of *Eimeria* parasites found after examination of the samples are listed in Figure 2. Five species of *Eimeria* (viz. *E. acervulina, E. tenella, E. maxima, E. brunetti* and *E. mitis*) were identified with different indices. *Eimeria acervulina* followed by *E. tenella* were the predominant species infecting chickens on the farms visited (32.05% and 26.92%, respectively). Statistically, the most prevalent *Eimeria* spp. was *E. acervulina* (*p* < 0.05). The morphometric index values of the oocysts isolated are presented in Table 3.

**FIGURE 3:**
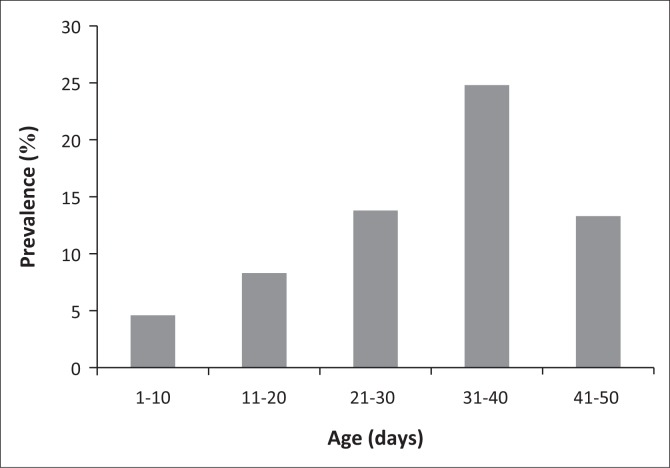
Prevalence of coccidiosis by age groups.

A significant association between the age of the chicken and the incidence of the coccidiosis (*p* < 0.05) was observed. Higher prevalence of coccidiosis was recorded in chickens in the age group 31–40 days (24.8%), followed by 21–30 days and 41–50 days (13.8% and 13.3%, respectively) and lower prevalence in the age group 11–20 days (8.3%), followed by 1–10 days (4.6%) (Figure 3).

Out of the 78 chickens scarified by cervical dislocation, post-mortem examination revealed a lesion of coccidiosis. We observed ballooning and petechiae on different intestinal portions. Hypertrophy of the intestine was seen in some portion of the intestines. Moreover, the caecal portion was found to be haemorrhagic. The mean OPG, sites and lesion scores of coccidiosis cases are shown in Table 2. The McMaster counting technique was used in this study to determine the oocyst burden in the samples. The OPG values recorded in the positive litter and intestinal content samples examined were found to be mostly infested by *Eimeria* oocysts in some cases.

**TABLE 2:** The mean oocysts per gram, sites and lesion score of *Eimeria* spp. at broiler chicken farms in Bejaia province.

Species	Intestinal site	Lesion score	Mean OPG (± SD) of positive samples (×10^3^oocysts/g)
*E. acervulina*	Duodenum and Jejunum	2–3	17.68 ± 2.70
*E. tenella*	Caecum	1–3	8.36 ± 1.86
*E. maxima*	Small intestine	1–2	1.88 ±2.78
*E. brunetti*	Ileumand colon	1	12.10 ± 3.61
*E. mitis*	Small intestine	-	5.16 ± 1.52

OPG, oocysts per gram; SD, standard deviation.

**TABLE 3:** Biometric characteristics of oocysts of different species of the genus *Eimeria* isolated in Bejaia province.

Species	Oocyst							Morphometric index
	Length			Width				
	±SD *µ*m	Min	Max	±SD *µ*m	Min	Max
*E. acervulina*	18.4 ± 3.94	15.7	20.3	14.5 ± 2.4	11.2	18.9	1.27
*E. tenella*	22.7 ± 2.4	20.5	25.3	18.9 ± 2.9	17.1	22.3	1.20
*E. maxima*	30.4 ± 3.5	27.9	34.5	22.8 ± 3.9	18.6	26.4	1.33
*E. brunetti*	23.4 ± 2.8	20.6	26.3	18.8 ± 2.4	16.9	21.6	1.24
*E. mitis*	18.2 ± 2.5	16.4	21.1	15.7 ± 3.6	11.6	17.8	1.15

Min, minimum; Max, maximum; SD, standard deviation.

Microscopic lesions resulted in epithelial necrosis and atrophy of the intestinal villi. Complete destruction of the epithelium and villi associated with haemorrhage were also observed. Histopathological examination revealed various changes in the tissue sections of both the caeca and the small intestine compared to those of the non-affected birds. These changes included a loss of epithelial tissue and congestion of the blood vessels.

## Discussion

The present study was conducted to evaluate the coccidiosis prevalence and to identify the species causing the parasitic disease in broiler chickens in Bejaia province. The results obtained revealed that the breeding broilers were infested by a variety of *Eimeria* species. The overall prevalence of coccidiosis was 54.28% (19 out of 35). In another survey conducted in the Blida area of northern Algeria, a similar prevalence of coccidiosis (55%) was reported (Triki-Yamani & Bachir Pacha 2010). This rate is low compared to investigations in Ethiopia (71.7%) (Dinka & Tolossa 2012), Saudi Arabia (80%) (Al-Quraishy et al. 2009) and Nigeria (87.4%) (Lawal et al. 2016). However, it is higher than the 11.4% and 14.0% reported respectively by Grema et al. (2014) and Adamu et al. (2009) in Nigeria. Moreover, Nematollahi et al. (2009) and Mwale and Masika (2011), Adang and Isah (2016), and Muazu et al. (2008) recorded a rate almost similar to the results of the present study in the north-west of Iran (55.9%), in South Africa (41.4%) and in Nigeria (42.7% and 52.9%), respectively. The variation in previous investigations might be attributed to different factors such as sampling periods, geographic area and climatic conditions (Lawal et al. 2016). The high prevalence value observed in the present work could originate from poor management practices in broiler breeding such as leaking water, accumulated faeces, poor hygiene, low ventilation and a high population density (Guinebert & Penaud 2005).

Based on the shape, measurements, number of oocysts and the site of infection, five species (i.e. *E. acervulina, E. tenella, E. brunetti, E. maxima* and *E. mitis*) were identified. *Eimeria acervulina* and *E. tenella* were the most prevalent species, with rates of 32.05% and 26.92%, respectively. Recently, *Eimeria* oocysts were isolated from poultry farms in north-east Algeria. The suspension contained *E. acervulina* and *E. maxima* as detected using morphometry and Polymerase Chain Reaction (PCR) methods (Djemai, Mekroud & Jenkins 2016). Similar findings of high prevalence of coccidiosis in chickens have been reported by various researchers (Amare, Mengistu & Nazir 2012a; Adang & Isah 2016; Adhikari, Gupta & Pant 2008; Agishi, Luga & Rabo 2016; Gadelhaq, Rafa & Aboelhadid 2014; Gharekhani, Dehkordi & Mohammadali 2014; Hadipour et al. 2011; Kaboudi, Umar & Munir 2016; Kumar et al. 2015; Sharma et al. 2015). Our results support reports from the literature in various countries that the identified species of *Eimeria* are widespread in broiler chickens.

Highly pathogenic species of *Eimeria* are responsible for the death of the chicken through haemorrhagic lesions that lead to blood loss and electrolyte balance (Adhikari et al. 2008; McDougald 2008; Williams et al. 2009). According to McDougald and Fitz-Coy (2008), *E. acervulina* is the most frequently encountered subclinical coccidiosis agent in commercial poultry. The two *Eimeria* species (viz. *E. acervulina* and *E. tenella*) observed in the present study have the highest reproductive potential (Williams et al. 2009). This considerable rate of *Eimeria* oocysts indicates that the failure to control the parasitic disease using chemoprophylaxis might be because of misuse of coccidiostat, which could induce the development of long-term resistance to anti-coccidial drugs (Hadipour et al. 2011; Zhang et al. 2013). The existence of genetic variation in resistance to coccidiosis infection among breeds and strains was also reported (Ashenafi et al. 2004).

The age of the chickens is considered as a major factor in the prevalence of coccidiosis infection. *Eimeria* spp. can cause infection in all ages (Badran & Lukesouna 2006; Sharma et al. 2015). Higher prevalence of coccidiosis at the age of 32–46 days might be associated with the presence of another immunosuppressive disease, such as Gumboro (Hachimi et al. 2008; Lanckriert et al. 2010; McDougald & Steve 2008). However, the results of this study showed that the age group of chickens 32–46 days was more alarming than the age group of 5–28 days. Many investigations have reported that younger chicks are more susceptible to natural infections than older ones (Ahmed et al. 2003; Al-Quraishy et al. 2009; Amare, Worku & Negussie et al. 2012b). Bachaya et al. (2012) observed that the prevalence of infection increased among younger chicks (60%) compared with older chickens (37%). Note that three steps are necessary for coccidian infestation in the litter of the farms, including a growth phase (21–28 days), an infestation peak (28–35 days) and a disintegration phase over 35 days.

Coccidiosis affects the host in several ways, depending on the tissue preference of the parasite involved and the number of oocysts ingested in the initial infection (Conway & McKenzie 2007; Smith & Sherman 2009). In this study, the infection intensity, measured as the total number of oocysts per gram of litter (14.96%) and intestinal contents (32.05%), had a high infection level (> 15 × 10^3^ oocysts). This is in agreement with a previous literature report (Lunden et al. 2000). De Gussem (2007) reported that the OPG count in faeces or litter has a poor relation to the impact of the parasite on the performance of a flock. The high number of OPG could be related to the resistance of *Eimeria* species in chicken. Intensive breeding is an additional factor that favours propagation of the coccidiosis disease (Badran & Lukešová 2006). Therefore, coccidiosis severity is positively correlated with the charge of ingested oocysts. The pathogenicity of coccidia depends on the species involved, the number of oocysts ingested and the host’s immune competence (Dakpogan et al. 2012).

Avian coccidiosis is classified into intestinal and caecal forms. These intestinal pathologies are the cause of the reduction in the absorption function of the mucosa affecting the small intestine by *Eimeria* species. In case of severe infections, caecal coccidians, namely *E. tenella* or *E. necatrix*, cause massive haemorrhages and anaemia (Conway & McKenzie 2007; Zhou et al. 2006). In the current study, the microscopic examinations of the affected caeca and small intestines showed severe tissue damage, which resulted in a histological lesion characterised by the disappearance of the surface epithelium and necrosis of the villi in some samples. Histopathology changes were indicative of the inflammation reaction brought on by parasitic infection with the *Eimeria* species, which has irritating effects on the intestines. Moreover, we noticed atrophy of the enterocytes lining the intestinal villi of the mucosa because of the presence of *Eimeria* spp. on site (Yakhchali & Tehrani 2007). Adamu and colleagues (2013) recorded a change in the histopathology of broilers caused by *E. tenella* and *E. brunetti*. The findings of this study are similar to those reported by Adamu et al. (2013) and Kawahara et al. (2014), who also recorded irregularity of the intestinal architecture affected by coccidiosis in chickens. The protozoan parasites of the genus *Eimeria* multiply in the intestinal tract and cause tissue damage, which results in mortality, interruption of digestive processes or nutrient absorption, reduction of weight gain and increased susceptibility to other disease agents (Yegani & Korver 2008).

## Conclusion

This is a first report on the prevalence of *Eimeria* species in broiler farms in Bejaia province, Algeria based on conventional methods. Our findings showed that five pathogenic *Eimeria* species in chickens occurred in Bejaia province, with *E. tenella* and *E. acervulina* being the most abundant. This study demonstrated that the coccidiosis is an omnipresent parasitic intestinal disease. It could strongly decrease the production performance in broiler chickens. Coccidiosis may be an important factor in the economic losses of the poultry farms in this region.
